# Use of Acute Psychiatric Hospitalisation: A Study of the Factors Influencing Decisions to Arrange Acute Admission to Inpatient Mental Health Facilities

**DOI:** 10.3389/fpsyt.2021.696478

**Published:** 2021-06-28

**Authors:** Rajan Nathan, Mark Gabbay, Sean Boyle, Phil Elliott, Clarissa Giebel, Carl O'Loughlin, Pete Wilson, Pooja Saini

**Affiliations:** ^1^Cheshire and Wirral Partnership NHS Foundation Trust, Chester, United Kingdom; ^2^NIHR CLAHRC NWC, Liverpool, United Kingdom; ^3^Institute of Population Health Sciences, University of Liverpool, Liverpool, United Kingdom; ^4^Health Education North West, Manchester, United Kingdom; ^5^School of Psychology, Liverpool John Moores University, Liverpool, United Kingdom

**Keywords:** mental health, admission, inpatient, healthcare services, psychiatric care, decision-making

## Abstract

**Background:** Human decision-making involves a complex interplay of intra- and inter-personal factors. The decisions clinicians make in practise are subject to a wide range of influences. Admission to a psychiatric hospital is a major clinical intervention, but the decision-making processes involved in admissions remain unclear.

**Aims:** To delineate the range of factors influencing clinicians' decisions to arrange acute psychiatric admissions.

**Methods:** We undertook six focus groups with teams centrally involved in decisions to admit patients to hospital (crisis resolution home treatment, liaison psychiatry, approved mental health professionals and consultant psychiatrists). The data were analysed using qualitative thematic analysis.

**Results:** Our analysis of the data show a complex range of factors influencing decision-making that were categorised as those related to: (i) clinical and risk factors; (ii) fear/threat factors; (iii) interpersonal dynamics; (iv) contextual factors.

**Conclusions:** Decisions to arrange acute admission to hospital are not just based on an appraisal of clinical and risk-related information. Emotional, interpersonal and contextual factors are also critical in decision-making. Delineating the breadth of factors that bear on clinical decision-making can inform approaches to (i) clinical decision-making research, (ii) the training and supervision of clinicians, and (iii) service delivery models.

## Introduction

The extensive empirically based literature evaluating the effectiveness of mental health interventions tends to focus on discrete “treatments” for specific clinical conditions (e.g., suicidal ideation) which may include psychological or pharmacological interventions [e.g., (1–4)]. However, the reality of mental health provision is that these well-defined and researched macro-interventions sit within a wider spectrum of activities, such as assessments, follow-up contacts, referrals, transfers of care and admissions (5). The macro-interventions aim to have an influence at broad levels such as national or institutional levels to review the linkages within several levels of a service or system. These activities for acute admission can influence outcomes by controlling access to discrete interventions (e.g., inpatient treatment), influencing patients' perceptions of the services, and serving as a means to offer micro-interventions (such as advice, education, and instillation of hope) (6). Despite the potential for these wider activities to impact on outcomes, they have not been subject to the same level of academic scrutiny as those elements of care more conventionally defined as treatment. Of the less studied interventions, acute admission to hospital is one of the most significant in terms of patient experience, micro-therapeutic opportunities, and resource utilisation (6, 10, 11, 23).

The limited literature that exists identifies crisis stabilisation, potential for harm and mental state acuity as key reasons for emergency admission [e.g., (7–11)]. The ways these decisions are made in practise are not well-understood and clinical experience points to significant variability. Predicting, managing and responding to pressures on the inpatient resource require greater clarity about the factors at play in real life decision-making at the point of acute admission. Furthermore, the development and application of technological support for decision-making relies on a thorough appreciation of how in-the-moment complex decisions are made (12).

Studies of decision-making in mental health settings have tended to concentrate on disorder-based and patient-based factors [e.g., (13–15)]. There have been some studies examining specific aspects of clinical decision-making such as shared-decision making (16, 17), the accuracy of decision-making (18), the role of human factors (19), and the influence of the way risk is framed (11). Nevertheless, there has been limited empirical analysis of how decisions are made in practise. One published study has specifically examined decision-making by mental health crisis team clinicians (20). However, a review of the literature did not identify any study that has specifically examined the wide range of factors influencing mental health practitioners' decision-making in relation to acute hospital admissions. Gaining a more in-depth understanding of these factors will inform approaches to clinical training and supervision of clinicians. Furthermore, service delivery models should take account of the way decisions are made in practise.

The objective of this study was to identify both clinical and non-clinical factors that clinicians consider when making decisions to admit patients to acute psychiatric units.

## Method

### Participants

The study was conducted in a large UK based NHS provider of community and hospital-based mental health services in the North West area of England. From a review of the service models and policies, four clinician-group types directly involved in decisions to acutely admit patients were identified: Crisis Resolution Home Treatment Teams (CRHTT), Liaison Psychiatrists, Approved Mental Health Professionals (AMHP) and Consultant Psychiatrists. All participants had been qualified and practising more than 2 years.

### Design

This study employed a qualitative approach using focus groups to explore the experiences of clinical staff making decisions for admitting mental health patients to hospital. Staff were invited to participate in focus group discussions. Six focus groups were conducted. The first was with the primary “gatekeepers” for the inpatient services, the CRHTT. Their remit was to assess and treat patients presenting with mental health problems in acute crisis from a specified catchment. The second group comprised Liaison Psychiatrists who were based in an acute hospital and responded to referrals from the hospital emergency department and inpatient wards. The third group was defined by the members having a specific non-medical role in the assessment and decision-making where legally mandated admission was considered necessary, the AMHP. The final group, Consultant Psychiatrists, become involved if there are particular complexities or involuntary detention is being formally considered.

### Procedure

SB introduced the study to the four eligible staff groups across the Trust. To reduce disruption to participants' clinical commitments, where possible focus groups were conducted at the end of a scheduled weekly review meetings.

The focus group facilitators comprised a clinician (SB), a clinical academic (RN), a service user representative (CO), a research manager (PE), and a university academic (PS). Semi-structured focus group schedules were designed with questions to facilitate the discussion about admission decisions. During the focus groups, participants were asked in general terms to talk about how they made decisions in relation to the acute admissions of patients to hospital. Groups were encouraged to talk in more detail about the range of reasons for their decisions. At the beginning of the focus groups, written informed consent was obtained from all participants. The focus groups lasted ~1 h. Focus Groups were recorded using a digital audio recorder and transcribed verbatim.

### Patient and Public Involvement and Engagement (PPIE)

CO, who had experience of being admitted to hospital, was involved from the inception of the study and contributed to the development and refinement of recruitment strategies, analysis of data, and dissemination plan and was leading on a further study exploring the views of other patients who had been admitted to inpatient wards.

### Data Analysis

**Figure 1:**
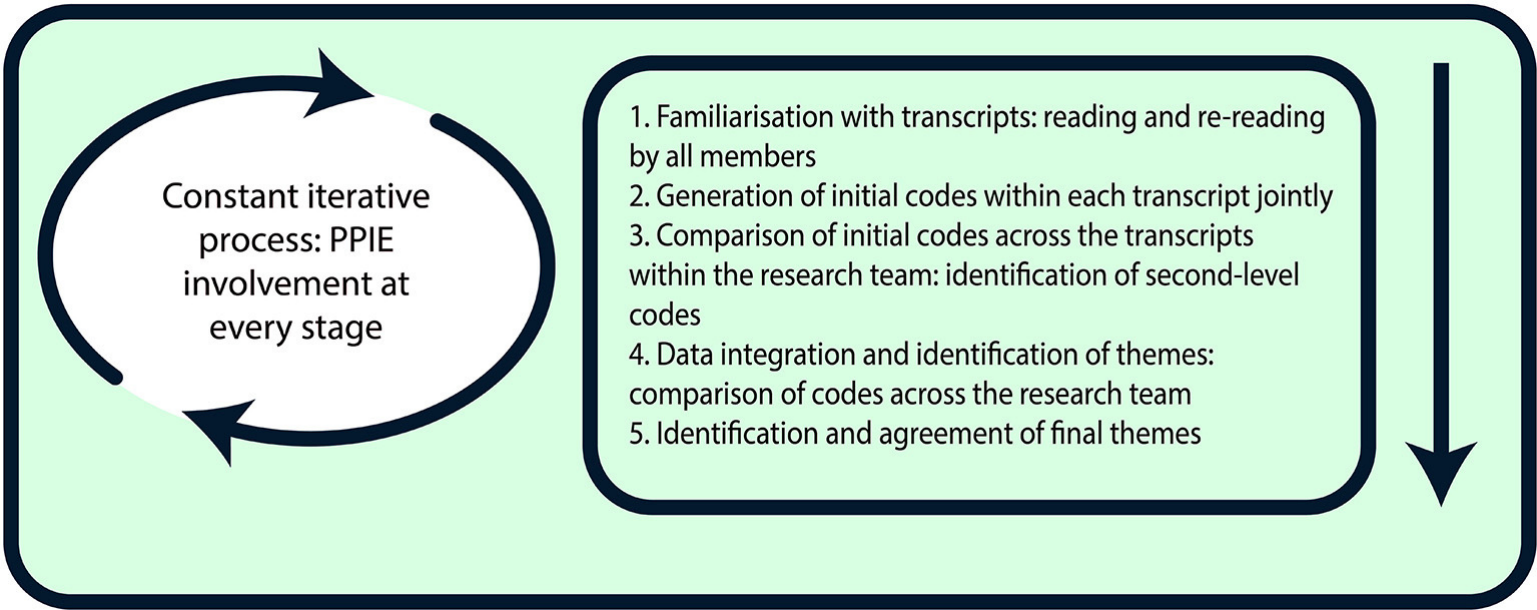
Thematic analysis process.

The analysis of all transcripts was conducted (Figure 1) and discussed by five members of the research team (SB, RN, CO, PE, and PS), each with different disciplinary backgrounds. The data were analysed following the principles of qualitative thematic analysis (21). The iterative coding process enabled the continual revision of themes until the final classifications of major themes were agreed by the team. The coding frame reflected our a priori interest in the theoretical concepts of transition and symbolic resources, and was also developed inductively from the entire data set. The frame helped categorise data in terms of the cultural (e.g., staff values), social (e.g., interpersonal relationships, organisational practises), and psychological (e.g., self-understandings as participants) aspects of decision-making (e.g., codes included “learning from past experiences,” “trusting professionals,” “reflecting upon oneself”). During repeated rounds frequent comparisons were made across codes and the interview data to develop, review, and refine themes (21) on the basis of the complementarity, convergence, and dissonance of ideas across data sources (22). All findings were then critically tested within the research group. Any disagreements were resolved by discussion.

### Ethical Consideration

Ethical approval was obtained from the Trust's R&D Department and University of Liverpool Ethics Research Committee prior to study commencement (Reference number: 2161). All participants were informed about the study via an invitation email that provided details of the study, a participant information sheet and the consent form.

## Results

Thirty-eight participants took part in focus groups between 26 June and 27 July 2017. Table 1 shows the breakdown of participants in each focus group.

**Table 1:** Participants who took part in each of the six focus group discussions.

**Focus group**	**Clinical group**	***N =* 38**
1	CRHTT^*^ (1)	7
2	CRHTT (2)	6
3	CRHTT (3)	5
4	Consultant psychiatrists	6
5	AMHP^**^	10
6	Liaison psychiatry team	4

* *Crisis resolution home treatment teams^1^*;

** *Approved mental health professionals^2^*.

**Figure 2:**
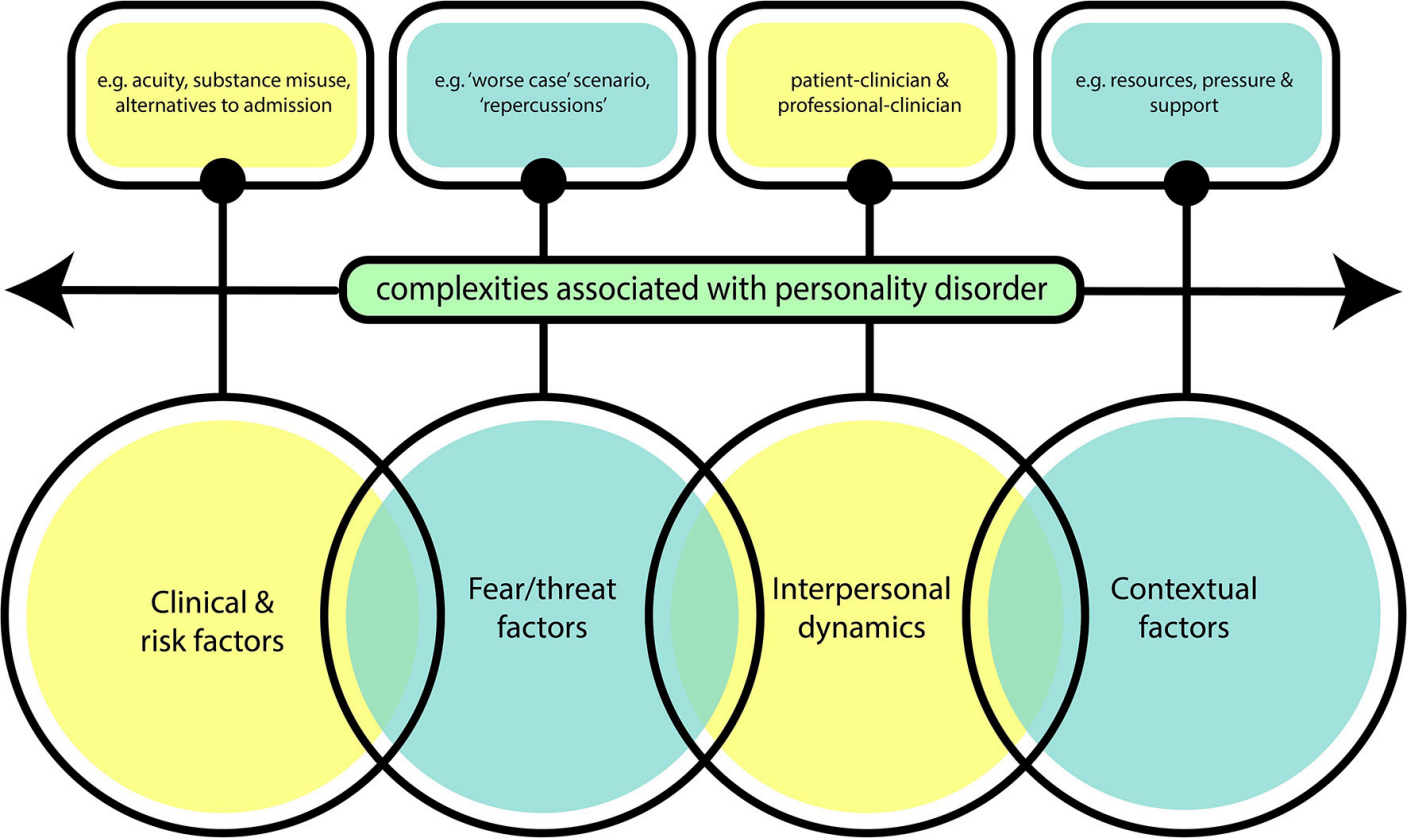
Figurative representation of key themes.

Four inter-related themes were conceptualised as reflecting the corpus of this material (Table 2 and Figure 2). The themes illustrated a more complex range of factors. The first theme *Clinical or Risk factors* theme encompasses issues that would be expected to influence decision-making such as acuity of patient clinical history, associated risks such as substance misuse and the viability of alternatives to admission. The second theme *Threat or fear factors influencing Clinicians* highlights the anticipation of negative evaluation of the clinician's practise in the event of a possible future serious untoward incident. The third theme *interpersonal dynamics* is related to the nature of the dynamic between the people involved in the decision that needs to be made. The fourth theme *contextual factors* identifies the circumstances of the context and assessment and other service issues such as resource availability. Each of these themes is developed below.

**Table 2:** Overview of themes and subthemes.

**Theme**	**Subtheme**
1. Clinical and risk factors	•Acuity of clinical history •Substance misuse •Alternatives to admission
2. Threat/fear factors	•“Worse case” scenario •Repercussions following decisions
3. Interpersonal dynamics	•Patient-clinician dynamic •Professional-clinician dynamic
4. Contextual factors	•The influence of resource availability •Pressure and lack of support for lone workers

### Clinical and Risk Factors

An emergent theme included those factors that would ordinarily be considered the basis of the decision to admit. These included the nature of the presenting clinical issues (e.g., “*clinical presentation*,” FG2; “*how they're presenting*,” FG3; ‘), the time course of those issues (e.g., “*what's been going on in the last few days, few weeks*,” FG6), and other factors relevant to the presentation (e.g., “*concordant with their medication. missing any of their secondary service appointments,”* FG6; “*under the influence of illicit substances or alcohol?*” FG6).

This theme also incorporated the notion of risk. Examples included “*risk factors that you identify in the assessment”* (FG2), whether “*someone had done something you know of a pretty serious nature to either put themselves at risk or someone else*” (FG5) and “*have they got forward planning [or] an intent to end their life*,” (FG2).

Related to both clinical and risk factors were considerations of whether the team can “*safely manage this person at home*” (FG3) or whether there are “*acute symptoms that cannot be managed in the community*” (FG2). In assessing this issue, practitioners “*look at a patient's care plan*” (FG1) and consider whether they “*live alone, have they got anybody to look after them*?” (FG6) and if there is “*evidence of carer breakdown, carer stress” (FG3)*.

Participants reported that decision-making involves consideration of potential risks of admission and whether other options have been considered:

“*admission … might be a really I guess dangerous experience for them if they become dependent on that environment*.” (FG1)“*for lots of reasons really it's better for the patient to be at home and rather than [hospital] it's not a great environment if you can avoid it … it's restrictive*.” (FG2)

“*people do view hospital as the way of keeping people safe and we know that that's not true, hospitals can't keep somebody safe*.” (FG3)“*we should have exhausted you know the all of you know collaborative options prior to making that decision to actually admit to hospital*.” (FG5)

There was a recognition that decision-making is subjective:

“*you can see it as inappropriate from the inpatient point of view but it may be appropriate from the community point of view … it's all perspective I don't think it's about what's appropriate or not appropriate*.” (FG4)

“*there are people's analytical sort of attempt to analyse the reason of admission and who are able to articulate it and put it in writing …. other people just intuition*” (FG4)

Patients with personality disorder were held to present a particular challenge for decision-making:

“*the [personality] disordered patients tend to be the ones that are the most challenging in terms of whether we send them home or we admit”… “the admissions aren't helpful to those disordered patients on the whole*.” (FG1)

“*some people with personality issues … say certain things to try and get into hospital and really you don't feel that they need to be in hospital er you feel that they probably won't act upon the things that they're saying and can easily be managed at home but unfortunately the things they are saying you've not got any choice sometimes to admit that person because you've not got the full MDT you're there on your own*.” (FG3)

“*diagnosis of EUPD [Emotionally Unstable Personality Disorder] quite frankly the hardest ones to deal with*.” (FG5)

Furthermore, patients with personality disorders seemed to be viewed more negatively and they were considered to present particular problems for in-patient management.:

“*personality disorders … are generally better if they are kept out of hospital you know because because their coping strategies are taken off them*.” (FG1)

“*If the patient's got a personality [sic] … admission generally isn't helpful to them. They generally start to escalate, self-harming behaviours when people start talking about discharge for instance*.” (FG6)

“*you're going to create an absolute bit of a nightmare environment on the ward because …., they all start to escalate their behaviours to match each other*.” (FG1)

“*some people you know personality issues could learn behaviours and then learn more maladaptive coping behaviours whilst they're on the ward*.” (FG3).

### Threat/Fear Factors

A prominent topic that emerged across the interviews was fear of future adverse outcomes for both a patient and the staff member making the decision to admit or not.

“*I sent him home but then I always sit back and I always think oh my god you know worse case again, worse case what happens if he does go out and he does harm somebody and they look at all the documentation*.” (FG1)

The fear was greater where the practitioner was the last professional to see the patient before the adverse incident:

“*there is fear now I mean I'm saying the word fear … so it makes you think … purely because based on the fact that … I'm the last person that's seen this patient*.” (FG2)

There was a particular focus on anticipated negative evaluation in the course of internal or external scrutiny processes such as being called to be a witness at a coroner's court if a patient was to die who was not admitted to hospital:

“*Usually there's a backlash from that …. like the threat of even coroner's court… we all dread it” (FG1)*

“*I think people also fear… the RCA [root cause analysis] process.” (FG3)*

The attribution of fault and the personal consequences were discussed across all of the focus groups and seemed to increase the potential of fear and guilt for participants:

“*if you send them away you're it's your fault he's dead*.” (FG2)

“*you know there is that worry about if things go wrong how will that look… what will be the repercussions from that*.” (FG3)

It was not only the anticipation, but also the experience of staff and of negative evaluation that was reported. Participants reminisced about previous events that had caused them distress and upset and how they had felt unsupported and on their own:

“*we were hounded and we had big meetings and we were told we'd failed this gentleman and the practitioner involved went through a horrendous time*.” (FG1)

“*cause I've been to coroner's and it was a quite horrendous 5 days event with barristers and everything it was awful, so everything I write I always look at it and think would he accept that*.” (FG2)

Participants felt that such experiences influenced their decision-making in admitting patients and thus making them more risk averse. The degree of support in the event of an adverse incident was referred to negatively:

“*you're under pressure to admit these people because of the risk and it's a case of like well I don't feel that they probably need to be in obviously but am I going to be backed up if something happens*.” (FG2).

### Interpersonal Dynamics

#### Patient-Clinician Dynamic

Participants gave descriptions of scenarios in which patients were overtly attempting to influence the practitioners' decision. They reported feeling pushed into a corner on occasion when patients threatened to harm or kill themselves if not admitted to hospital. They felt that some patients manipulated them into making a decision to admit them.

“*Sometimes the patient can push you in to a corner and say well if you send me home I'm going to kill myself*.” (FG1)

“*the line which can be quite powerful around what a person may say that they will now be driven to do*.” (FG3)

“*he was quite cute in how he was portraying I think he was sort of playing a bit of a game*.” (FG5)

“*the person will always go out of a lot of time will go out and do something to try and influence that. … and that can be anything nothing to do with mental health whatsoever*.” (FG6)

Participants referred to some patients having the expectation to be admitted once they had made that decision themselves. Some patients were perceived to sabotage their home treatment so that they would be admitted to hospital and others presumed that they could be admitted for long periods:

“*It's hard when someone's expectation is admission and sometimes they'll reluctantly agree to [home treatment] but you know … will sabotage it … and not engage*.” (FG1)

“*Public's preconception that we keep people in hospital for months on end and they think that that will solve everything and they don't realise that might only be a couple of days”* (FG6)

Another issue reported by participants to influence decisions to admit was questions about whether a patient was being truthful about their condition or their stated reasons for wanting admission:

“*Are they telling you the truth?*” (FG6)

“*We've had people trying to hide from criminal proceedings but obviously they keep that part of it all quiet*.” (FG6).

#### Professional-Clinician Dynamic

Participants described the influence of other professionals on the decision-making dynamic, particularly when finding themselves in awkward positions; for instance when patients had already been informed that they would be admitted to hospital prior to their assessment taking place. Scenarios such as these sometimes influenced the clinician to make a decision to admit a patient, when perhaps this might not have been the outcome if an assessment had been conducted without raising a patient's expectations first:

“*a big factor is the patient's expectations from other professionals*.” (FG2)

“*I turned up, there was two practitioners for the CMHT and they had already said he was to be admitted and told his girlfriend and told him*.” (FG2)

“*really difficult because those expectations are raised*.” (FG3)

The pressure to admit a patient by other staff, family members or patients themselves was discussed. In some cases, participants would feel influenced and pressured into making the decision to admit a patient. Reflecting on the fear previously discussed, participants reported they would admit patients to avoid the criticism and backlash if anything were to go wrong in the future rather than due to the clinical need of a patient:

“*everybody around them is screaming at you to admit or you feel as if you know if you don't as we've said and something does happen adverse happens to them that you're just going you're going to get all that sort of criticism of why you didn't admit so there is a big pressure*.” (FG1)

“*you might have pressure from A&E staff who have already done an assessment and put high risk needs admission erm you've got the family shouting at you that they need admission so there's lots of other factors if it's the middle of the night sending them out at 3 o'clock in the morning when they say they're going to kill themselves there's those threats, there's all those things that influence your decision*.” (FG2)

These examples further illustrate how participants' may be influenced when another professional commits to a decision that can cause problems if it is then not adhered to:

“*once other professionals have seen them and already made the decision that they feel they need to be in hospital you are kind of sometimes backed into a corner*.” (FG2)

“*it's difficult to deviate from the prevailing consensus view particularly when it's about risk or high risk*.” (FG5).

### Contextual Factors

#### The Influence of Resource Availability

Participants highlighted the importance of resource availability when considering whether to admit patients. If there was no bed availability in the local or nearby inpatient wards then a decision to manage a patient at home may have been made, whereas if there was a bed available the same patient may have been admitted:

“*People feel a lot of pressure because there's less inpatient beds*.” (FG2)

The lack of beds can then also increase the pressures on community teams who are then expected to follow up more patients within community settings and those at higher risk (who would have been admitted if a bed was available) will need to be monitored more often; thus adding more hours to their workload:

“*Our community teams are really struggling with their capacity as well*.” (FG3)

Additionally, there was a belief expressed by participants that patients with personality disorder had come to distract resources away from those with “serious mental illness” [SMI]:

“*we didn't look after people with [personality] disorders. … We just looked after people with mental illness … but a lot of CMHT's time now is taken up with is taking up with managing and those people that are escalating their behaviours and seeking that constant attention that that constant because they don't know how to manage themselves and they're always seeking outside support… the SMIs are generally on the peripheries aren't they*.” (FG1).

#### Pressure and Lack of Support for Lone Workers

The pressure of working alone in the community or on night shifts within both hospital and community settings were raised as pertinent issues for participants. Night shifts were reported as the most difficult times of day to conduct assessments as often staff were making decisions on their own whereas during the day they may have had the opportunity to liaise with colleagues or other support networks:

“*you're like a lone practitioner ‘cause you know if you've got like a few assessments waiting*.” (FG2)

“*it would have been a different decision from day time with other services available, with other support*.” (FG3)

Furthermore, participants felt that for some cases there was not enough time to reflect and discuss assessments with others about whether to admit patients or not and this was thought to be an important factor that may have influenced the outcome of their decisions:

“*the time to reflect to communicate if you think about what prevents an admission is the ability to step back think about it, discuss it with your colleagues er when you're having to work 100% all the time you don't get that time to be able to reflect with colleagues*.” (FG4).

## Discussion

This study is the first to directly examine both the clinical and non-clinical factors that influence mental health clinicians' decision-making in relation to acute psychiatric admissions. It addresses an important topic that has been relatively understudied, considering the frequency of psychiatric admissions required to manage symptom exacerbations and crises. Discussions with a representative group of decision-makers found a wide range of factors that fell within four broad themes. Similar to previous findings (6, 10, 11, 20), the clinical/risk factor theme encompasses issues that would be expected to influence decision-making (e.g., the clinical history, associated risks and the viability of alternatives to admission). This study demonstrated that clinician decision-making was influenced by wider factors that are rarely explicitly examined in studies of psychiatric admissions.

The critical element of the second theme (threat/fear factors) was the consequences of a serious adverse event (particularly suicide or serious violence) following their assessment. Prior experiences and pre-existing beliefs by clinicians have been reported to affect hospitalisation decisions (6). In this study, these were predominantly described as either the personal or vicarious past experience of negative evaluation of practise after an adverse event or a concern about a future investigation process and the anticipation of resulting negative evaluation of the clinician's practise. More research is needed on how past experiences influence clinician's future decision-making processes.

The third theme (interpersonal dynamics) was manifest in the form of clinicians attributing intent to the patient (e.g., assuming the patients' account was deliberately modified to influence the clinician to act in a way that otherwise they would not have done) which they then found made decision-making more difficult. If patients or other clinicians had prior expectations about the need for admission, this could also interfere with the assessment and decision-making. An additional element of the interpersonal dynamic theme was the pressure from others (e.g., other professionals or family).

The final broad theme (contextual factors) included general service capacity factors such as the reduced availability of inpatient beds. Contradictory findings are available for the influence of bed availability with older studies suggesting this has no significance influence (23) but more recent studies identifying this as a relevant factor (6, 24). The increased pressures on community services as well the specific context (e.g., availability of peer support, lone working and the time of day) in which the clinician undertook the assessment influenced decisions to admit patients.

This study highlighted that an assessing practitioner or clinician is not just making a decision on hospitalisation. They are simultaneously deciding if not hospital, then where else may the patient be treated, such as via home treatment, community mental health support or in primary care. The decision-making process seems to be about how to get the most appropriate support for the person at that specific point in time. Similar to Lombardo et al. (20) this study found that patient needs was the primary driver behind decisions but that heuristics also played a key role in decision-making for clinicians.

Running across all four themes was the particular relevance of personality disorder to decision-making. In line with previous studies (25–27), the clinical assessment of patients with personality disorder (as opposed to those with mental illness) was considered more challenging. This study suggests tensions, negative attitudes and possibly a negative bias of staff toward this patient population. Previous studies have highlighted factors that influence attitudes which include service setting, practitioners' level of experience and the absence of specific training to enhance an understanding of personality disorder (27).

The findings of this study suggest training and supervision of mental health clinicians making decisions about patients should not just focus on how to elicit and interpret case-based clinical and risk-related information. Consideration should also be given to how to deal with wider influences which include fears of the consequence of future adverse events, pressures on decision-making from a range of sources and the relevance of contextual factors. The findings also support the need to take a whole systems approach to the way decisions are made. The study highlights how current approaches to scrutinising past practise after serious incidents can have an adverse effect on future practise. The theoretical framework “mindlines” may be a useful starting point to consider where these influences sit within the overall decision-making processes, albeit based around knowledge management in practise (28, 29).

### Strengths and Limitations

Since this paper mainly references the content of focus groups with mental health clinicians, it is limited to their perspective. However, a strength of the study is the number of participants (*n* = 38) and representation being sought from across the region and four different mental health disciplines. Previous studies have been limited to collecting data from one team (20). Future studies may also explore if predefined roles of teams influence their decision-making. Associated affect (30) may lead to greater reporting of certain factors (e.g., threat/fear factors which by definition are associated with negative affect) and under-reporting of other less affect-laden scenarios. The use of focus groups rather than 1:1 interviews may have inhibited some participants from disclosing sensitive issues. It is not possible from this research to know the degree to which the identified factors actually do influence decisions in real time. The participants all worked within the same organisation and the role of organisation-specific cultural influences on decision-making cannot be not determined. This study focused on the perspective of professionals and it is essential future research also includes patients' experiences of decisions being made about them.

## Conclusions

This study reveals that clinical decision-making at the point of hospital admission entails more than just making sense of clinical and risk-related data. Clinicians are faced with managing wider complex intra- and inter- personal factors that have the potential to interfere with the way the decision is made. Uncovering the breadth of relevant factors can contribute to a more informed approach to research into clinical decisions, to the training and supervision of clinicians, and to service delivery models.

## Data Availability Statement

The raw data supporting the conclusions will be made available by the authors, in a format that ensures the anonymity of the participants.

## Ethics Statement

The studies involving human participants were reviewed and approved by University of Liverpool Ethics Research Committee (Reference number: 2161). The patients/participants provided their written informed consent to participate in this study.

## Author Contributions

RN, SB, CO'L, and PS conceived of and designed the research. SB and RN collected the data. SB, RN, CO'L, and PS analysed the data. RN and PS wrote the manuscript. All authors contributed to the article and approved the submitted version.

## Conflict of Interest

The authors declare that the research was conducted in the absence of any commercial or financial relationships that could be construed as a potential conflict of interest.

## Footnotes

^1^Crisis Resolution Home Treatment Teams undertake urgent assessments of patients in acute crisis and provide signposting to appropriate services and/or short-term support.

^2^Approved Mental Health Professionals are authorised to carry out certain duties which are critical to assessments and admissions of patients under the Mental Health Act.
