# Erratum: Use of Acute Psychiatric Hospitalisation: A Study of the Factors Influencing Decisions to Arrange Acute Admission to Inpatient Mental Health Facilities

**DOI:** 10.3389/fpsyt.2021.745801

**Published:** 2021-08-19

**Authors:** Frontiers Production Office

**Affiliations:** Frontiers Media SA, Lausanne, Switzerland

**Keywords:** mental health, admission, inpatient, healthcare services, psychiatric care, decision-making

Due to a production error, AMHP (Approved Mental Health Professionals) was incorrectly labelled as AMPH (Approved Mental Health Practitioners). A correction has been made to the section **Methods**, subsections **Participants** and **Design**, and in **Table 1**.

The publisher apologizes for this mistake. The original article has been updated.

